# Geographic Distribution, Genetic Variability and Biological Properties of Rice Orange Leaf Phytoplasma in Southeast Asia

**DOI:** 10.3390/pathogens10020169

**Published:** 2021-02-04

**Authors:** Socheath Ong, Gilda B. Jonson, Matteo Calassanzio, Soriya Rin, Cheythyrith Chou, Takao Oi, Ikuo Sato, Daigo Takemoto, Toshiharu Tanaka, Il-Ryong Choi, Chhay Nign, Sotaro Chiba

**Affiliations:** 1 Department of Crop Protection, Faculty of Agronomy, Royal University of Agriculture, Ministry of Agriculture, Forestry and Fisheries, Dangkor District, Phnom Penh 370, Cambodia; osocheath@rua.edu.kh; 2 Nagoya University Asian Satellite Campuses Institute—Cambodian Campus, Royal University of Agriculture, Dangkor District, Phnom Penh 370, Cambodia; soriya.kcj@gmail.com (S.R.); I.Choi@irri.org (I.-R.C.); 3 Rice Breeding Platform, International Rice Research Institute, Los Baños, Laguna 4031, Philippines; G.Jonson@irri.org; 4 Department of Agricultural and Food Sciences, University of Bologna, Viale G. Fanin, 40127 Bologna, Italy; matteo.calassanzio@gmail.com; 5 Renolab Good Laboratory Practice, A Tentamus Company, Via XXV Aprile, San Giorgio di Piano, 40016 Bologna, Italy; 6 General Directorate of Agriculture, Ministry of Agriculture, Forestry and Fisheries, Tuol Kork, Phnom Penh 370, Cambodia; thyrith72@gmail.com (C.C.); chhay.ipm@gmail.com (C.N.); 7 Department of Plant Production Sciences, Graduate School of Bioagricultural Sciences, Nagoya University, Chikusa-ku, Nagoya 464-8601, Japan; oitaka@agr.nagoya-u.ac.jp (T.O.); isato@agr.nagoya-u.ac.jp (I.S.); dtakemo@agr.nagoya-u.ac.jp (D.T.); totanaka@agr.nagoya-u.ac.jp (T.T.); 8 International Rice Research Institute—Korea Office, National Institute of Crop Science, Wanju-Gun 235, Jeollabuk-Do, Korea

**Keywords:** rice orange leaf phytoplasma, *Candidatus* phytoplasma asteris 16SrI-B subgroup, genetic divergence, DAPI staining, scanning electron microscopy, storage starch accumulation

## Abstract

Rice orange leaf phytoplasma (ROLP) causes clear orange to yellowish leaf discoloration and severe stunting in rice seedlings. The ecological and biological characteristics of ROLP are largely unknown because the disease has not widely caused serious problems in rice cultivated areas, thereby leading to the low accumulation of research data. However, in the past decade, the disease became a threat to rice production, particularly in South China and India; it has also been recognised in other Asian countries, such as Vietnam, Thailand and the Philippines. Here, we observed the occurrence of ROLP in paddies of the Southeast Asian counties (Cambodia, Vietnam and the Philippines) and found that the isolates in the Philippines and Vietnam were monophyletic, while those in India, Thailand and Cambodia were more diverse, suggesting their potential origins. In Cambodia, it was revealed that following polymerase chain reaction (PCR) detection, the known ROLP-insect vectors, *N. virescens* Distant and *Recilia dorsalis* Motchulsky, were ROLP-positive, indicating their roles in pathogen dispersal. Moreover, fluorescent and scanning electron microscopy revealed the intensive accumulation of the phytoplasma in phloem tissues and massive accumulation of storage starch in vascular bundle sheath and parenchyma. Altogether, this study illustrated the genetic variability of global ROLP isolates and the pathogen’s biological impact on rice tissue.

## 1. Introduction

Phytoplasma is a group of bacteria that are unculturable in vitro due to them lacking a cell wall, localised in the phloem of diverse host plants (more than 1000 species), vectored by phloem-feeding insects and taxonomically classified in the genus “*Candidatus* Phytoplasma”, which accommodates 44 species separated based on 16S ribosomal DNA (rDNA) sequences [1,2]. Rice (*Oryza sativa*) is known to be affected by two phytoplasmas: rice yellow dwarf phytoplasma (RYDP), causing rice yellow disease [3,4], and rice orange leaf phytoplasma (ROLP), causing rice orange leaf disease (ROLD) [3,5,6]. The RYDP belongs to “*Ca.* P. oryzae” (16SrXI group), while the ROLP is classified in “*Ca.* P. asteris” (16SrI group) [2,3]. *Ca.* P. asteris also includes important pathogens of diseases, such as oenothera aster yellows, onion yellows, rhus yellows, mulberry dwarf and paulownia witches’ broom [7]. Today, the detection of ROLP mainly relies on nested polymerase chain reaction (PCR) and sanger sequencing of the amplicon with approximately 1.2 kbp [8,9,10].

The RYDP-infected rice exhibits leaf chlorosis, profuse tillers with stunting and results in the failure of grain production, which are distinct from ROLD symptoms [3]. The rice yellow disease by RYDP has been reported in many rice-growing countries of East and Southeast Asia since its first record in Southwestern region of Japan in 1910 [11,12]. RYDP is known to be vectored by several green leaf hopper (GLH) species such as *Nephotettix nigropictus* Stål, *N. cincticeps* Uhle and *N. virescens* Distant (Hemiptera: Cicadellidae) [3,12]. These insects also transmit viruses including rice dwarf virus, rice tungro spherical virus and rice tungro bacilliform virus [13,14,15].

In contrast, ROLD is represented by orange leaf colouration starting from the leaf tip downwards [3]. The ROLP-affected plants are often moderately stunted, leaves roll inwards and cause senescence, reduced tiller numbers are observed and eventually significant grain yield loss is caused [16,17]. The degree of these symptoms varies according to the infection time, plant condition and environment—for example, leaf discoloration was observed from yellow to golden or deep orange, and the ROLP infection at seedling stage is lethal, but plants can survive when the infection occurs at matured stages [18].

Since the first recognition of ROLD in 1960, phytoplasma associated with ROLD was widely observed and reported in Thailand, Malaysia, Sri Lanka, Philippines, China and other Asian countries by the 1980s, which was confirmed by electron microscopy [19,20]. These did not cause severe problems at that time because diseased rice plants were sporadically found in paddies, despite significant damages and death of infected plants [19]. However, in the decade of the 2010s, ROLD and ROLP 16S rDNA sequences were increasingly reported in China, Thailand, Vietnam, India and the Philippines [3,18,21,22,23], suggesting a global expansion of the disease. ROLP is reported to be transmitted by zig-zag leafhopper (ZLH), *Recilia dorsalis* Motchulsky (Hemiptera: Cicadellidae) [19]. This was recently supported by PCR detection of ROLP in field-collected insects in India and China [6,24] and by transmission tests [6]. Additionally, ROLP transmission by alternative insect vectors (GLH), *N. cincticeps* and *N. virescens,* that mainly disperse in temperate and tropic zones, respectively, have been evidenced [6,18]. 

Given the expansion of ROLD in Asian countries, ROLP is considered as a potential threat to global rice production. In Southern China, heavily infested rice fields were found in Guangdong province in 2015, and the disease spread to surrounding areas (Guangxi and Hainan provinces) between 2016 and 2018 [6,25,26]. In Thailand, a nation-wide survey was conducted, and it revealed the wide-spread occurrence of ROLD in the country [23,27], and in the Philippines, a recent survey revealed the re-emergence of the disease on different islands [18]. Although the cases have been reported, the mechanism of ROLP infection in the rice ecosystem and rice plants is largely unknown. To understand a part of these, in this study, we conducted a further survey regarding ROLD and the sequencing of ROLP 16S rDNA in three countries—Vietnam, the Philippines and Cambodia—to understand the genetic variations of global ROLP isolates and their geographic distributions. Furthermore, the population density of insect vectors in Cambodian paddies was monitored, and the rate of ROLP acquisition by insects was assessed. In addition, altered plant condition of ROLD-affected rice was analysed microscopically. Obtained results provide an insight into the nature of the pathogen, *Candidatus* P. asteris, in rice. 

## 2. Results

### 2.1. Field Survey of ROLP in the Philippines, Vietnam and Cambodia

**Figure 1:**
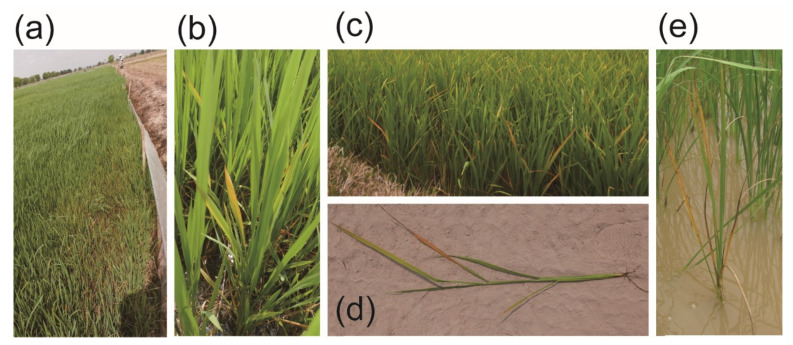
Field observations of potential rice orange leaf phytoplasma (ROLP)-infested paddies in Cambodia. (**a**) and (**b**) Yellowing rice plants with impaired growths. (**c**,**d**) Rice plants showing orange discoloration directed downward from the tip. (**e**) A rice plant showing progressed leaf senescence and death.

**Figure 2:**
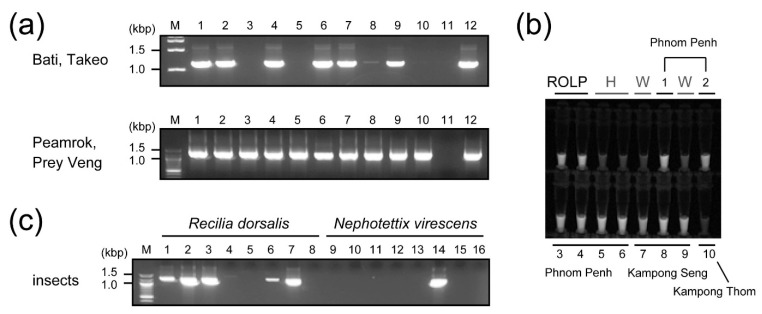
Molecular detection of ROLP in rice samples collected from paddies showing typical rice orange leaf disease (ROLD) symptoms and sweep-collected insects. (**a**) Agarose gel electrophoretic image of nested polymerase chain reaction (PCR) products amplified with ROLP-specific primer pairs. Results of rice samples from Takeo and Prey Veng provinces are shown. M, DNA ladder marker; 1–12, field-collected rice samples. (**b**) Loop-mediated isothermal amplification (LAMP)-mediated detection of phytoplasma in the tested rice samples. ROLP, positive control plants; H, healthy rice plants; W, negative control with water; 1–10, field-collected rice samples. Origin of samples is stated above or below the sample numbers. (**c**) PCR detection of ROLP from the insects, *Recilia dorsalis* (zig-zag leafhopper) and *Nephotettix virescens* (green leafhopper). M, DNA size marker; 1–16, field-collected insect samples.

From 2015 to 2019, we conducted field surveys in the Philippines (four provinces) [18], Cambodia (five provinces) and Vietnam (two provinces). Some paddies were seen to be affected by ROLP (Figure 1a), as rice plants exhibiting yellowish (Figure 1b) or golden leaf discoloration were sporadically present (Figure 1c,d). Causal correlation between these symptoms and ROLP was roughly assessed by nested PCR or loop-mediated isothermal amplification (LAMP) or both (Figure 2a,b), and the following sequence analyses. ROLP was detected from all provinces where field surveys were conducted, and the phytoplasma detection rate of analysed samples had varying percentages in each country (48.7–88.2%), suggesting the wide occurrence of ROLD in Southeast Asian countries (Table 1). In most of the sites where ROLD-suspected rice samples were collected, ROLP was present and detected at a rate of over 50%, except for Angkorchey district, Takeo province in Cambodia (3/11, 27.3%) and Tan Hong district, Dong Thap province in Vietnam (5/15, 33.3%) (Table 1). Although severe damages have not been observed in the paddies in general, rice plants in a paddy of Svay Rieng province in Cambodia showed lethal symptoms (Figure 1e).

### 2.2. Phylogenetic Analysis of ROLP Based on 16S rDNA Sequences

**Figure 3:**
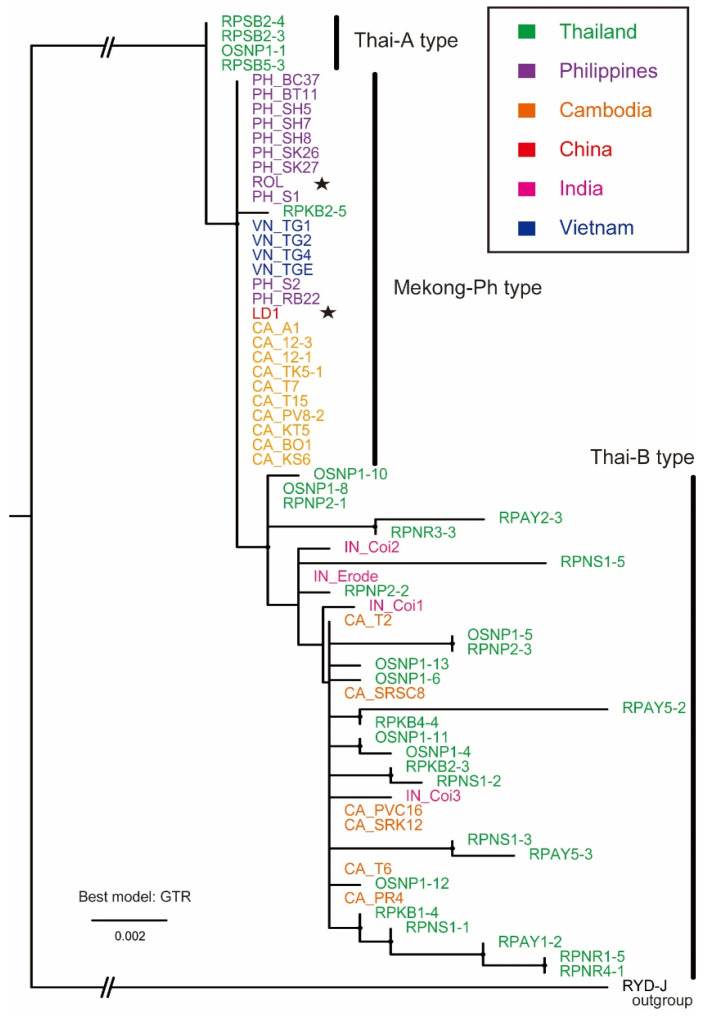
Maximum-likelihood phylogenetic analysis of 16S ribosomal DNA (rDNA) sequences of global ROLP isolates. The ROLP 16S rDNA sequences obtained by the nested PCR products with the 2nd PCR primer set (R16F2n and R16R2) were phylogenetically analysed with ROLP sequences deposited in GenBank. The origin of isolates is colour-differentiated as follows: Thailand (green), the Philippines (violet), Cambodia (orange), China (red), India (pink) and Vietnam (blue). Stars indicate the first isolates with 16S rDNA sequence from the Philippines (ROL) and with draft genome sequence from China (LD1). Accession numbers of used sequences are listed in Supplementary Table S1.

Representatives of nested PCR products, as shown in Figure 2a, were subjected to Sanger sequencing, and obtained sequences were analysed accordingly by basic local alignment search tool (BLAST) searching and were found to be of the ROLP 16S rDNA. Selected ROLP isolates for sequence and phylogenetic analyses are shown in Table 1 and Supplementary Table S1. Multiple alignments of these DNA sequences were created exclusively from partially determined sequences and subjected to a maximum-likelihood phylogenetic analysis. The result is visualised as a mid-point rooted tree with the J strain of rice yellow dwarf phytoplasma (RYD-J) as an out group in Figure 3. Publicly available sequences of Thai isolates (green) were the most divergent, but formed a single large cluster with all ROLP isolates; however, they were separated into three subgroups, (1) a probable ancestor-like group (Thai-A type), (2) a potential eastwardly transferred group (Mekong-Ph type) and (3) a possible progeniture-like group with genetic variability (Thai-B type). The Thai-A type consists of only four isolates from Thailand. Interestingly, all 11 Philippines isolates and all four Vietnam isolates are grouped with a singly reported Chinese isolate sequence. These sequences are identical to each other and other partial sequences of isolates from Dong Thap province, Vietnam, and the rest of those in the Philippines (Table 1). These are most closely related to a Thai isolate RPKB2-5 from Kanchanaburi and form the Mekong-Ph type subgroup. This subgroup includes 10 out of 16 Cambodian isolates as well. In the Thai-B type subgroup, six Cambodian and four Indian isolates are scattered in the cluster, formed by 25 out of 30 Thai isolates. 

### 2.3. Insect Population Dynamics and Detection of ROLP from Field-Collected Insects

**Figure 4:**
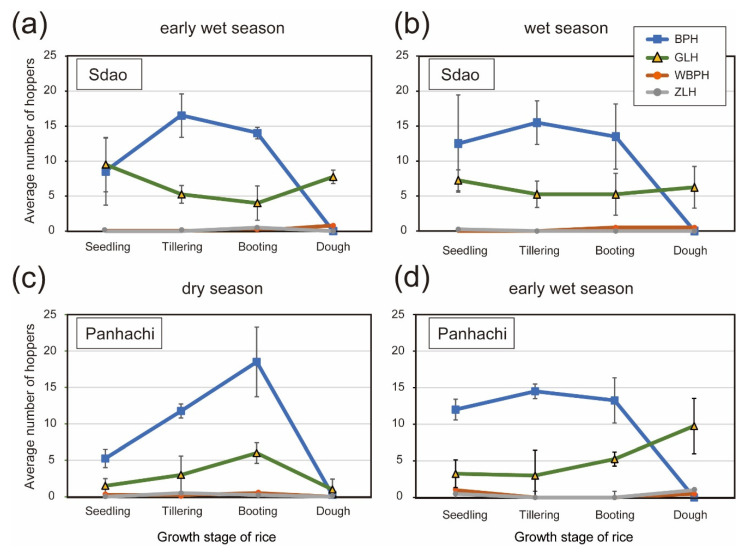
Total number of selected leafhoppers and planthoppers in different locations and seasons in Cambodia. (**a**) Total counts of brown planthopper (BPH), white backed planthopper (WBPH), zig-zag leafhopper (ZLH) and green leafhopper (GLH) at seedling, tillering, booting and dough stages of rice in a paddy located in Sdao, Cambodia, during the early wet season. (**b**) Insect count in the same paddy as (**a**) during the wet season. (**c**,**d**) Total insect count in a paddy located in Panhachi, Cambodia, during the early wet (**c**) and dry seasons (**d**).

Zig-zag leafhopper (ZLH, *R. dorsalis*) and green leafhoppers (GLH, *N. cincticeps* and *N. virescens*) are known ROLP vectors [6,14,18]. In the previous study, *N. virescens* was found abundant in the paddies of the Philippines; however, *N. cincticeps* was not observed [18]. Similarly, *N. virescens* but not *N. cincticeps* was found in Cambodian paddies where rice samples were collected. A low population of ZLH was found in the same paddies, regardless of seasons and rice crop stages (Figure 4). Alternatively, in all seasons, the GLH (*N. virescens*) population was generally high in the seedling, tillering and booting stages of rice growth. Brown planthoppers (BPHs) were the most abundant in the monitored sites.

By nested PCR, the ecological roles of ZLH and GLH (*N. virescens*) for ROLP transmission in Cambodia were assessed. Although ZLH was less present in the paddies, insects carried ROLP at a high rate (62.5–100%) (Figure 2c, Table 2). In contrast, while the GLH (*N. virescens*) population was high in the paddies, the ROLP detection rate was not high as that of ZLH (16.7–42.9%) (Figure 2c, Table 2). These results suggest that both insects have vital roles in the spread of ROLP in the rice ecosystem of Cambodia.

### 2.4. Phloematic Accumulation of ROLP

**Figure 5:**
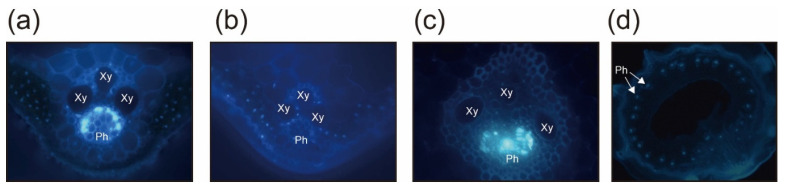
Fluorescent microscopic observation of 4’,6-diamidino-2-phenylindole (DAPI)-stained thin sections of ROLP-infected rice plants. (**a**,**b**) Transversal section of leaf tissue stained with DAPI from the ROLP-infected (**a**) and healthy (**b**) rice plants. (**c**,**d**) DAPI-stained transversal section of stele tissue of root from the ROLP-infected rice with a high (×200) (**c**) and low (×50) (**d**) magnitudes. Ph, phloem; Xy, xylem vessel.

The behaviour of ROLP in its lifecycle in rice and insects is largely unknown. 4′,6-Diamidino-2-phenylindole (DAPI) staining of phytoplasma genomic DNA and observation under a fluorescent microscope was applied to develop an instant observation protocol for ROLP [10]. Using the rice plants that were ROLP-positive following nested PCR, thin sections of rice leaves were prepared and stained with DAPI. By this conventional method, ROLP was presumably detected in rice phloem as bright-blue fluorescence (Figure 5a) that was absent in the control specimen (ROLP-free healthy rice plants) (Figure 5b). Additionally, this fluorescence was observed in root phloem (Figure 5c). Notably, most of the phloem in the same root tissue exhibited a fluorescent signal, suggesting that ROLP could be propagated and spread throughout the plant (Figure 5d).

### 2.5. Histological Observation of ROLP-Infected Rice

**Figure 6:**
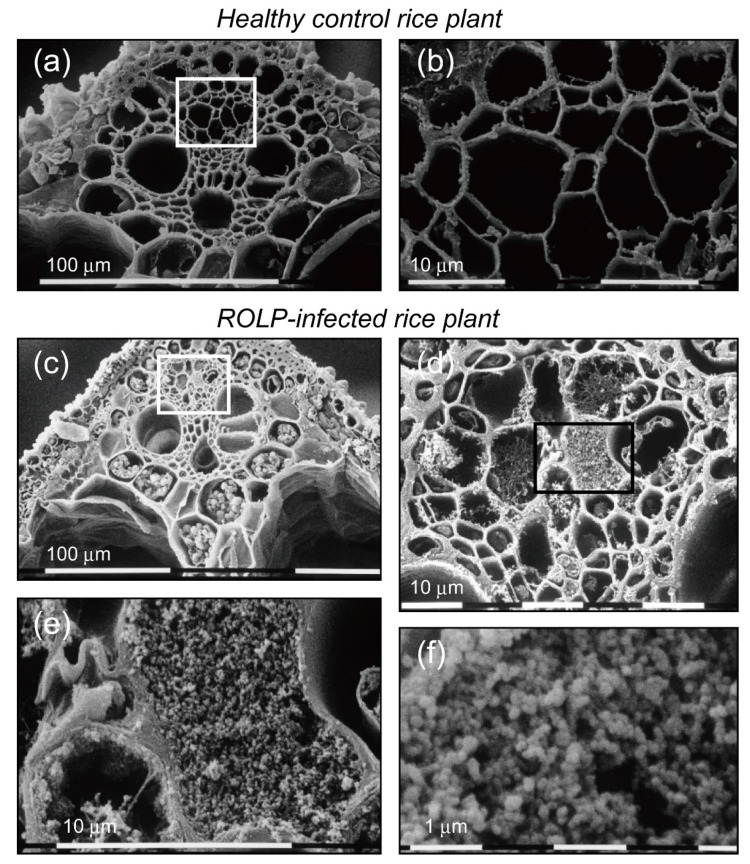
Scanning electron micrographs of the ROLP-infected rice leaves. (**a**) Thick section of healthy rice leaf. (**b**) Enlarged rectangular image in (**a**) showing the phloem. (**c**) Thick section of ROLP-infected rice leaf. High quantity of storage starch was found in vascular bundle sheath and parenchyma. (**d**) Enlarged image of rectangle in (**c**) showing the phloem. (**e**) Enlarged image of rectangle in (**d**) showing a phloem with compaction of small particles. (**f**) Small particles in the phloem of ROLP-infected leaf. Black and white bars at the bottom are scale bars of indicated sizes.

In the ROLP-infected rice plant, drastic physiological changes occur. Thick sections of rice leaf blade were prepared and observed under a scanning electron microscope (SEM) to assess the changes in plants. The major difference between healthy and diseased rice plants is the massive accumulation of starch-like granules in the ROLP-infected rice leaf (Figure 6). In the vascular bundle sheath and parenchyma of the ROLP-infected rice, compacted, amorphous blobs of possible storage starch were observed (Figure 6c), while a very low amount was seen in healthy plants (Figure 6a). Similarly, in the phloem’s enclosed view, particle compaction was observed only in the ROLP-infected rice (Figure 6b,d). The sizes of these particles appeared to be very small (100–200 nm in diameter), and this range agrees with the general size of the phytoplasma (80–800 nm in diameter) (Figure 6e,f). These obvious changes are associated with ROLD symptoms, such as leaf discoloration, growth inhibition and fast senescence.

## 3. Discussion

### 3.1. Global Population Structure of ROLP Based on Currently Available 16S rDNA Sequences

**Figure 7:**
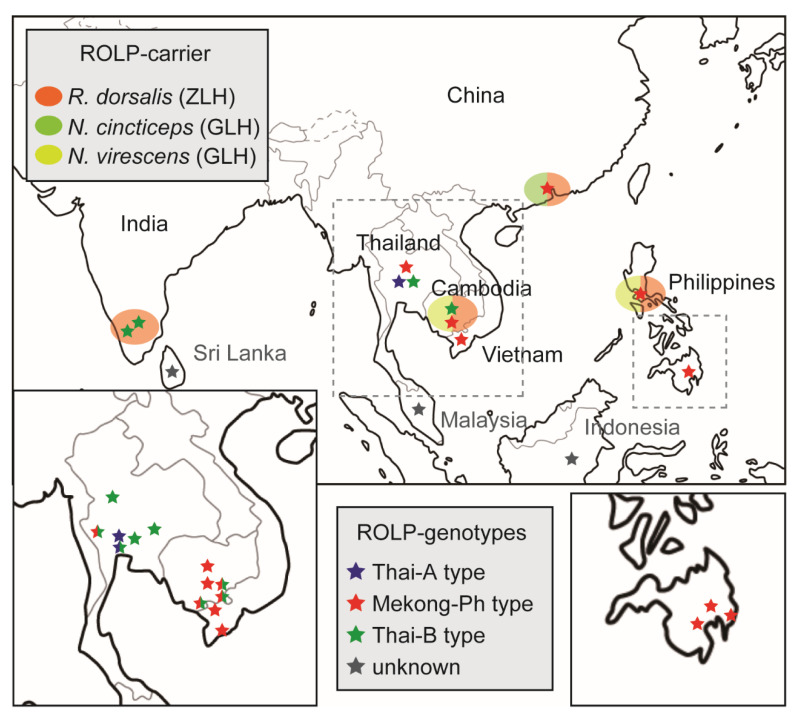
Geographic distribution of three ROLP subgroups in Asian countries. Presence of phylogenetically separated three subgroups (genotypes) of ROLP are differentiated by stars with blue (Thai-A type), red (Mekong-Ph type) and green (Thai-B type) in each country in the main map. Those of previously reported ROLP with no genetic information are shown by a grey star. Enclosed mappings of ROLP distribution in Vietnam/Cambodia/Thailand and the Philippines are shown; each star in these enclosed maps represents different provinces and stars with mixed colours indicate detection of different ROLP subgroups in a province. Confirmed ROLP-carrying insects are shown by circles with different colours as indicated.

The current study detected a considerable number of ROLP-infected rice plants (236 samples out of 295 plants tested) in the Philippines, Vietnam and Cambodia from 2015 to 2019 (Table 1) and confirmed that these are associated with insect vectors ZLH and GLH in Cambodia (Table 2). A series of surveys were triggered by the first observation of ROLD by our team in Laguna, the Philippines, in 2015 and that in Svay Rieng, Cambodia in 2016. At this stage, ROLP in Cambodia was expected to have migrated from Vietnam [22]. Together with the reported ROLP 16S rRNA gene sequences from Thailand, India, Vietnam, the Philippines and South China [3,18,21,22,23,28], those of the Philippines, Vietnam and Cambodia analysed in this study were phylogenetically assessed for the first time (Figure 3). It was found that isolates in India, Cambodia and Thailand were more diverse than those in other countries, and thus these may be considered as autochthonous genotypes of ROLP in these areas or international translocation-resultant isolates. Although further analysis is required, it is anticipated that the potential origin of ROLP is Thailand because of its rich genetic diversity (Figure 3). Additionally, probable spread of a single population in Cambodia, Vietnam, China and the Philippines was assumed based on the distribution of the Mekong-Ph type ROLP in these countries as shown in Figure 7 (red stars). Indeed, the evolutional relationship between the Chinese and Indian isolates is far apart; the disease outbreaks in these countries may not be attributed to the genetic commonality of ROLP. To our knowledge, ROLP can be found commonly in the paddies of Asian countries; thus, the pathogen is expected to be more broadly present in rice cropping countries today. To further understand the genetic variations of ROLP, disease surveys must be conducted more widely together with genotyping of the pathogen by sequencing of 16S rDNA and multilocus house-keeping genes [26,29,30] or even by genome-wide comparisons.

### 3.2. Paddy Leafhopper and Planthopper Populations and the Transmission of ROLP in Southeast Asia

ZLH carried ROLP at high rates in the diseased rice fields in Cambodia, but the population density of ZLH was very low throughout the rice growth stages and in different cropping seasons (Table 2, Figure 4). GLH had relatively low rates of ROLP detection; however, large numbers of these were found in the paddies. Although the insect’s population structure has not been assessed in the Philippines, similar trends of less ZLH and many GLH in rice fields were observed previously [18]. This also agrees with the situation in South China where another GLH (*N. cincticeps*) of the temperate region played an essential role in the pathogen transmission [6]. Moreover, while the acquisition rate of this GLH species (16.7–55.3%) was comparable to that of the GLH in the tropics (*N. virescens*) (16.7–42.9%) (Table 2), the germ-carrying rate of ZLH in China (31.7–61.6%) was lower than those in Cambodia (62.5–100%) (Table 2). This fact may reflect different affinity levels of the ZLH biotypes to ROLP. In fact, ZLH in Cambodia seemed much smaller in size than it is generally known. To this regard, an investigation into the affinity of ROLP surface proteins with each vector is required. VmpA of Flavescence dorée phytoplasma (16SrV-C and-D) was associated with *Spiroplasma citri* transmission ability [31], and the antigenic membrane protein (Amp) of onion yellows phytoplasma (16SrI) formed Amp–microfilament complexes in insects that defined transmissibility [32]. Altogether, we hypothesized that both ZLH and GLH are important vectors of ROLP in Cambodian rice fields, which is probably the case in Vietnam and the Philippines [18]. 

In addition, it was observed that BPH was the most abundant and the primary pest in two paddies in Cambodia (Figure 4). The high population density of BPH might be associated with the opportunistic transmission of ROLP; therefore, two additionally monitored insects were subjected to ROLP detection by nested PCR. As a preliminary result, only one of tested BPHs and white backed planthoppers (WBPHs) was ROLP-positive, suggesting the low acquisition rate of ROLP by these insects, although their transmission ability is currently unknown (Supplementary Table S2). These observations raise awareness of the requirement of further investigation on field insects for ROLP acquisition and transmission. 

Small holder cultivated Cambodian rice fields and the timing of rice planting among those farmers is not uniform, being situated in the concomitant presence of rice plants at different growing stages in a small area [33,34]. The quick drop in the population density of BPH at the dough stage (Figure 4) is expected to be a result of BPH migration to other rice fields with a feasible environment [35]. If BPH has ROLP transmission ability, it will contribute to the distant spread of the disease. In contrast, the GLH density at the dough stage was maintained during the humid season (early wet and wet seasons) (Figure 4). This ecological characteristic of GLH might contribute to the constant maintenance of ROLP in the rice ecosystem, as for the tropics; farmers keep cropping rice three, up to four, times in irrigated areas. Thus, controlling the GLH population before rice planting may be a useful measure of ROLP control.

### 3.3. Biological Properties of ROLP in the Host Rice Plants and Future Research Prospects

The molecular mechanisms of infection, spread and pathogenicity of ROLP in rice plants are largely unknown. Akin to other phytoplasmas, ROLP was observed in the phloem by transmission electron microscopy [6,14]. In this study, highly accumulated ROLP as a DAPI-stained genome in the phloem of rice leaves and roots was confirmed (Figure 5). This led to the speculation that the microparticle compaction in the phloem observed under the SEM (Figure 6d–f) was of ROLP bodies, which agrees with the sizes of known phytoplasma cells [1]. PCR detection of ROLP from rice samples is very effective as it is generally detectable in the first round of nested PCR (1.5 kbp product). This, therefore, suggests a high propagation of ROLP in the phloem, which is correlational with microparticle compaction in the phloem. It is interesting to quantitatively compare phytoplasma accumulations between different phytoplasma or host plants. Technically, the DAPI staining/fluorescent observation to detect phytoplasma is a useful approach for understanding the systemic distribution of the pathogen in plant tissues [10,36]. The combination of this and development of artificial inoculation cycle in the laboratory is highly desired.

Phytoplasma infection causes drastic phenotypic changes in host plants—many examples report physiological changes upon infection. For example, mulberry yellow dwarf phytoplasma (16SrI group) infection changed the content of host metabolites, including carbohydrates, amino acids, organic acids and others [37]. It should be noted that the phytoplasma also attenuates starch digestion ability by the downregulation of α- and β-amylase genes, whereas photosynthesis was inactivated, resulting in higher starch accumulation levels in plant [37]. In this regard, as observed in Figure 6c, ROLP infection caused high accumulation of starch-like granules in vascular bundle sheath and parenchyma. To further understand the biological impact of ROLP infection in rice, transcriptomic and metabolomic analyses need to be addressed. 

Other microbes and viruses may affect the host rice or ROLP once a mixed infection is established. The detection of a DNA virus, rice tungro bacilliform virus (RTBV), was attempted using the available rice DNA samples. As previously observed [18], the virus was not detected in most ROLP-positive rice samples tested in Cambodia. However, those from Bati, Takeo province, contained a few positive and faintly positive samples (Supplementary Figure S1). Interestingly, samples showing a strong signal of RTBV were negative for the ROLP detection, whereas faint signals were found in a few ROLP-positive samples. In tomato, tomato big bud phytoplasma (16SrII-D) and tomato yellow leaf curl virus were found to be antagonistic to each other in terms of their accumulation levels and symptom expressions [38]. As rice tungro viruses rely on the insect vector GLH for their transmission, it is highly interesting to investigate whether these viruses act counteractively or synergistically against ROLP in rice plants and insect vectors. 

## 4. Materials and Methods

### 4.1. Field Survey of ROLP-Infested Rice Paddies

A rice field survey was conducted from 2015 to 2019 in Svay Rieng, Prey Veng, Takeo, Kampong Thom, Kampong Seng provinces and Phnom Penh in Cambodia, Tien Giang and Dong Thap provinces in Vietnam and Mindanao and Laguna provinces in the Philippines. A portion of this survey was previously reported [18]. Paddies with ROLD-suspected rice plants were targeted and leaf or whole plant samples as well as insects were harvested or collected upon farmers’ agreement. Plant and insect samples were processed for DNA extraction or stored at −68 °C.

### 4.2. DNA Extraction, Nested PCR and LAMP

Rice and insect samples were homogenised in liquid nitrogen, and genomic DNA fractions were extracted using a Cetyl trimethyl ammonium bromide (CTAB)-mediated conventional extraction method [18], DNeasy plant mini kit or DNeasy blood and tissue kit (Qiagen, Hilden, Germany) by following the manufacturer’s instructions. 

In total, 50 ng of purified DNA was subjected to nested PCR with well-designed universal primer sets: P1 and P7 for the first round PCR and R16F2n and R16R2 for the second round PCR as previously described [6,18]. The first PCR program was 94 °C for 2 min; 30 cycles at 94 °C for 30 s, 52 °C for 30 s, and 72 °C for 2 min; 72 °C for 10 min. The second PCR program was 94 °C for 2 min; 30 cycles at 94 °C for 30 s, 50 °C for 30 s and 72 °C for 2 min; 72 °C for 10 min. The second PCR amplicons with lengths of 1.2 kbp were examined by 1% agarose gel electrophoresis.

According to the provided protocol, a phytoplasma universal detection kit (Nippon Gene, Tokyo, Japan) was used for phytoplasma detection by LAMP, and confirmed by nested PCR later on for ROLP confirmation. 

### 4.3. Sequencing and Phylogenetic Analysis

PCR products were gel-purified with a Wizard SV gel and PCR clean-up system (Promega, Madison, WI, USA) and sent to Macrogen (Seoul, South Korea) or Eurofin (Tokyo, Japan) for sequence analyses, with newly designed primers: ROLPseq-intF1 (5′-GAAACTTAAAGGAATTGAC-3′), ROLPseq-intF2 (5′-TAACTATGTGCCAGCAG-3′) and ROLPseq-intR (5′-TTATAGCATCACAATGTTG-3′). Obtained sequence data were processed using Genetyx (Genetyx, Tokyo, Japan) and assembled. Each reconstructed sequence (1201 bp in length, excluding primer region) was confirmed by BLAST search. The multiple sequence alignments were constructed in MAFFT v.7 online (https://mafft.cbrc.jp/alignment/server/ (accessed on 4 February 2021)) [39] and converted into Phylip format. A maximum-likelihood phylogenetic analysis was performed using PhyML v.3.0 (http://www.atgc-ontpellier.fr/phyml/ (accessed on 4 February 2021)) along with the best-fit models calculated using smart model selection in PhyML (http://www.atgc-montpellier.fr/sms/ (accessed on 4 February 2021)) [40]. Obtained tree information was visualised in FigTree and enhanced with Adobe Illustrator.

### 4.4. Insect Population Analysis

In Sdao, Prey Veng province and Panhachi, Kampong Thom province, in Cambodia, insect population analyses were conducted either during the early wet, wet or dry seasons at four different cropping stages—seedling, tillering, booting and dough stages—in 2017 and 2018. Briefly, the insect pests in rice fields were collected with a sweep net (30–40 cm in diameter) from one-quarter of about 1000 m^2^ paddy and counted, thereby indicating four data replications in the paddy. From the collection data sets, the numbers of ZLH, GLH, BPH and WBPH were extracted. In the Sdao site, assessments were conducted in the early wet season (17 April–12 July 2017) and wet season (17 September–28 November 2017). In the Panhachi site, the same assessment was performed in the early wet season (28 March–20 June 2017) and in the dry season (29 October 2017–25 January 2018). 

### 4.5. Microscopic Observation

Healthy and ROLF-infected rice plants were screened by nested PCR. DAPI staining of the rice tissues was performed by following the protocols of phytoplasma study [41,42]. Root and leaf tissues were used for specimen preparations. Tissues were fixed in 5% formaldehyde in 0.1 M phosphate buffer, pH 7.0 for 30 min. Then at each step, they were washed twice in phosphate buffer, pH 7.0, for 3 min. Free-hand sections between 20 and 100 μm thickness were stained with 0.001% DAPI in 0.01 M phosphate-buffered saline, pH 7.4, for one hour, mounted in water or glycerine and examined under a BX53 fluorescence microscope (Olympus, Tokyo, Japan) using the U excitation method (U-FUW/U-FUN). 

Specimens for SEM were prepared from the rice leaf bundle. Circular micro-sections with 1–2 mm thicknesses were excised from the middle portion of the leaf blades, and immediately fixed in 5% glutaraldehyde in 0.1 M phosphate buffer (pH 7.2). After rinses in 0.05 M phosphate buffer, the sections were subjected to dehydration in ethanol (10%, 25%, 50%, 75%, 95%) in distilled water and in absolute ethanol. A subsequent treatment in isoamyl acetate was performed before dehydrating the sections by K850 Critical Point Drying (Quorum Technologies Ltd., Laughton, England). The specimens were covered with a layer of gold particles using K500 sputter (Quorum Technologies Ltd., Laughton, England) at 40 mA and observed by SEM S510 (Hitachi, Tokyo, Japan) with an accelerating voltage of 15 kV. 

## Figures and Tables

**Table 1:** Summary of field survey of ROLP in Cambodia, Philippines and Vietnam during 2015–2019 and representative ROLP isolates of those 16S rRNA gene were sequenced.

Country	Year	Province	District ^a^	Detection ^b^	Sequenced Isolates ^c^	Note
Cambodia	2016	Svay Rieng	Svay Chrum	13/20	CA_A1 ^d^, CA_B1 ^d^,	This study
					CA_12-1, CA_12-2 ^d^,	
					CA_12-3	
	2017	Takeo	Treang	21/21	CA_T7, CA_T15	This study
		Takeo	Bati	15/20	CA_T6	This study
		Takeo	Angkorchey	3/11	CA_T2	This study
		Prey Veng	Peamrok	18/20	CA_PR4	This study
		Prey Veng	Prey Veng City	20/20	CA_PVC16	This study
		Svay Rieng	Svay Chrum	18/20	CA_SRSC8, CA_SRK12	This study
	2018	Kampong Thom	Steung Sen	3/3	CA_KS6	This study
		Kampong Thom	Santuk	2/4	CA_KT5	This study
	2019	Phnom Penh	Dangkor	5/6	CA_BO1	This study
		Prey Veng	Peamrok	4/4	CA_PV8-2	This study
		Takeo	Trumkok	13/14	CA_TK5-1	This study
	2016–2019	(Cambodia total)		135/163		Avg. detection rate: 82.8% ^e^
Philippines	2015	Laguna	Los Banos	2/2	PH_602 ^d^	This study
	2016	Laguna	Los Banos	2/2	PH_A1	This study
	2017	Laguna	Los Baños	49/60	PH_S1, PH_S2, PH_S9 ^d^	Jonson et al. (2020)
	2019	Davao del Sur	Hagonoy	8/8	PH_SH5, PH_SH7,	Jonson et al. (2020) ^f^
					PH_SH8	
			Matanao	2/2		Jonson et al. (2020)
		Davao del Norte	Sto Tomas	9/9	PH_SK26, PH_SK27	Jonson et al. (2020) ^f^
		Davao Oriental	Cabangcalan	10/10	PH_BC37, PH_BT11,	Jonson et al. (2020) ^f^
					PH_BT12 ^d^, PH_RB22	
	2015–2019	(Philippines total)		82/93		Avg. detection rate: 88.2% ^e^
Vietnam	2017	Tien Giang	Tan Phuoc	14/24	VN_TG1, VN_TG2,	VN_TG4 (Thi et al., 2016) ^g^
					VN_TG4, VN_TGE-1	
		Dong Thap	Tan Hong	5/15	VN_DT-13 ^d^, VN_DT-3 ^d^,	This study
					VN_DT-5 ^d^, VN_DT6 ^d^	
	2017	(Vietnam total)		19/39		Avg. detection rate: 48.7% ^e^
Cambodia, Philippines and Vietnam in total				236/295		Avg. detection rate: 80% ^e^

^a^ District or Municipality in the provinces are shown. ^b^ Number of ROLP-positive plants per number of total plants, which were examined using PCR or LAMP. ^c^ 1.2 kbp PCR fragments of 16S rRNA gene were sequenced and their accession numbers are listed in Table S1. ^d^ Partial sequence (>1.0 kbp) was obtained but not included in phylogenetic analysis (Figure 3). ^e^ Averaged detection rates in a country or in three countries are shown. ^f^ Sequence analysis was performed in this study. ^g^ Re-sequenced in this study.

**Table 2:** PCR-based ROLP detection from field-collected insects *Racilia dorsalis* and *Nephotettix virescens* in Cambodian paddies.

		*Racilia dorsalis* (ZLH)		*Nephotettix virescens* (GLH)	
Province	District	Detection ^a^	Rate	Detection ^a^	Rate
Takeo	Bati	11/11	100%	3/7	42.9%
Prey Veng	Peamrok	1/1	100%	1/6	16.7%
Prey Veng	Prey Veng City	5/8	62.5%	1/6	16.7%
Svay Rieng	Svay Chrum	4/5	80%		
(total detection number and averaged detection rate)		21/25 ^b^	84% ^c^	5/19 ^b^	26.3% ^c^

^a^ Number of ROLP-detected insects from the total number of tested insects. ^b^ Total detection number per tested plant sample number is shown. ^c^ Averaged detection rate is shown.

## Data Availability

The accession numbers for sequence data obtained in this study (MG712772, LC598903-LC598939) are listed in Supplementary Table S1.

## Supplementary Materials

The following are available online at https://www.mdpi.com/2076-0817/10/2/169/s1, Figure S1: PCR detection of Rice tungro bacilliform virus (RTBV); Table S1: List of 16S rDNA sequences of Rice Orange Leaf Phytoplasma (As of 10 August 2020); Table S2: The ROLP detection from field-collected insects *Nilaparvata lugens* and *Sogatella furcifera* in Cambodian paddies.
